# Frailty in an Adult Acute Hospital Population: Predictors, Prevalence, and Outcomes

**DOI:** 10.3390/ijerph21030273

**Published:** 2024-02-27

**Authors:** Rónán O’Caoimh, Laura Morrison, Maria Costello, Antoinette Flannery, Cliona Small, Liam O’Reilly, Laura Heffernan, Edel Mannion, Ruairi Waters, Shaun O’Keeffe

**Affiliations:** 1 Department of Geriatric Medicine, Mercy University Hospital, Grenville Place, T12 WE28 Cork City, Ireland; 2 Health Research Board Clinical Research Facility, University College Cork, Mercy University Hospital, T12 WE28 Cork City, Ireland; 3 Department of Geriatric and Stroke Medicine, University Hospital Galway, Newcastle Rd, H91 YR71 Galway City, Irelands.okeeffe@hse.ie (S.O.); 4 Department of Emergency Medicine, University Hospital Galway, Newcastle Rd, H91 YR71 Galway City, Ireland

**Keywords:** hospital, prevalence, predictors, frailty, comprehensive geriatric assessment, length of stay, re-admission, mortality, institutionalisation

## Abstract

Frailty is common among older hospital inpatients. While studies describe frailty prevalence in acute hospitals, it is usually based upon retrospective hospital-coded data or brief screening on admission rather than comprehensive geriatric assessment (CGA). Further, little is known about differences between pre-admission and current frailty status. Given this, we investigated the prevalence of pre-frailty and frailty among adult inpatients in a large university hospital after CGA. Of the 410 inpatients available, 398 were included in the study, with a median age of 70 years; 56% were male. The median length of stay (LOS) at review was 8 days. The point prevalence of frailty was 30% versus 14% for pre-frailty. The median Clinical Frailty Scale score pre-admission was 3/9, which was significantly lower than at review, which was 4/9 (*p* < 0.001). After adjusting for age and sex, frailty was associated with greater odds of prolonged LOS (odds ratio [OR] 1.7, *p* = 0.045), one-year mortality (OR 2.1, *p* = 0.006), and one-year institutionalisation (OR 9, *p* < 0.001) but not re-admission. Frailty was most prevalent on medical and orthopaedic wards. In conclusion, CGA is an important risk assessment for hospitalised patients. Frailty was highly prevalent and associated with poor healthcare outcomes. Frailty status appears to worsen significantly during admission, likely reflecting acute illness, and it may not reflect a patient’s true frailty level. The development of frailty clinical care pathways is recommended in order to address the poor prognosis associated with a diagnosis of frailty in this setting.

## 1. Introduction

Frailty is a common syndrome, predominantly affecting older people, that can be described as a multi-factorial, age-associated decline in the ability to manage stressors, which predisposes vulnerable, at-risk individuals to adverse healthcare outcomes [1]. These include prolonged hospital length of stay (LOS) [2,3], mortality [3], reduced functional independence [4,5], and nursing home admission after discharge [6]. Identification of those who are frail or at risk of becoming frail (pre-frail) [7] is an important public health issue at a population-level [8] and is included as a priority area for ageing policies in many countries [9]. Older adults and particularly those with frailty are high users of acute hospital care [10]. Frailty is associated with increased use of healthcare resources during an inpatient admission and overall higher hospital costs [11].

The prevalence of frailty is markedly higher in hospital than community samples [12], and it is estimated to be between 2 and 4 times higher there than among community-dwelling populations [3]. In acute care settings, systematic reviews and meta-analyses provide pooled prevalence estimates that vary from 34% [13] to 45% [12] for older hospital inpatients. The prevalence of more marked frailty, labelled as moderate to severe frailty, in hospital ranges even more widely between 14.3% and 79.6% [3]. Frailty, while present on most hospital wards [12], is not evenly distributed; frailty prevalence is estimated to be approximately 50% higher in older inpatients on acute medical wards compared to surgical wards [3].

Frailty status on admission to hospital can predict a range of adverse outcomes, including inpatient falls, delirium, pressure ulcers, and mortality [14]. Data from a systematic review and meta-analysis shows that frailty in this setting increases risk of death by 3.5 times [15]. It also predicts long-term adverse outcomes after hospitalisation [16]. Characterisation of frailty helps to facilitate better monitoring and can potentially provide opportunities to initiate preventative management and target limited healthcare resources appropriately [14,17,18]. Early identification in hospital is important for improving outcomes for older people by promoting selective cohorting to geriatric wards and targeted comprehensive geriatric assessment (CGA) [18,19], a multi-disciplinary diagnostic and therapeutic process that is well established as reducing mortality and institutionalisation when delivered in hospital [19]. This is particularly important for frail inpatients, whose care is often scattered throughout hospital wards under different medical and surgical specialties [12,20,21]. In surgery, for example, frailty screening is important for peri-operative care, and for identifying those at higher risk for anaesthesia and with greater requirement for post-op critical care [20].

While growing data are available on the prevalence of frailty in acute hospitals [12], they are usually based upon coded data [17,21], brief screening on initial admission via the emergency department (ED) [22], or analysis of ward- or specialty-specific data [23,24,25]. A recent scoping review highlighted that most studies examining frailty prevalence in hospital have, to date, been conducted in EDs, geriatric wards, and general medicine wards but found that only approximately half of studies used frailty-specific instruments and few (13%) confirmed independently whether patients were actually frail [26]. Most studies have attempted to measure frailty on admission to hospital and this was often determined by retrospective chart review rather than by formal assessment [26]. Even where frailty instruments were used, the majority of studies applied individual frailty screening or assessment instruments, with few conducting CGA to accurately determine an individual’s true (baseline) pre-admission frailty status [12]. Further, while routine data collection using coded data to generate frailty scores automatically for admitted patients enhances compliance with frailty screening [17], such approaches often have low diagnostic accuracy [27], suggesting that these studies may not be measuring true baseline frailty, but rather the effects of acute illness or multi-morbidity. Hence, intervention based on limited screening without further assessment with CGA to confirm frailty may be inappropriate, particularly as CGA is a limited resource. It may, in part, explain why studies examining interventions in hospitalised adults with frailty show marked heterogeneity with a recent systematic review identifying only one study that used frailty as an outcome [18].

While the optimal timing of frailty identification in hospitals is unclear, profiling patients as they are admitted to wards may be more appropriate than in the ED as many consider frailty screening to be challenging in this venue for several reasons, including lack of training, time, or space for ED staff [28,29,30,31]. Further, patients are usually acutely unwell, potentially impacting on the accuracy of frailty screening. Few, if any, studies have investigated the point prevalence of frailty and pre-frailty, based on CGA after admission, examining the impacts on outcomes up to one year after discharge, and to our knowledge none have done so in Ireland. Instead, such studies have mainly used screens as surrogates for diagnosis [15], without clear evidence that these instruments have high diagnostic accuracy rather than just good predictive validity in this setting. Understanding the epidemiology of true baseline frailty, independent of the apparent effects of multi-morbidity or intercurrent illness, is important for better characterising its impact and risk factors and the effectiveness of interventions. In an acute setting in an Irish and European context, such information will support the development of national and regional integrated frailty pathways for older people [32]. Hence, the aim of this study was to investigate the point prevalence, predictors, and outcomes associated with true baseline frailty and pre-frailty among all adult inpatients across an entire large university hospital in the West of Ireland, determining frailty using CGA as a gold standard measure.

## 2. Materials and Methods

### 2.1. Inclusion and Exclusion Criteria

This study was designed as a cross-sectional observational study. Consecutive inpatients aged >18 years of age who were an inpatient on a single day in May 2016 in a large, acute, and adult university hospital in the West of Ireland were eligible for inclusion. Specifically, we invited all patients who were included on the computerised hospital inpatient census record at 16.00 on the day of the study. This was performed to avoid overcounting if new admissions happened during the window of completing data collection. Those deemed medically stable by the attending nurse or doctor on the patients’ ward were approached and invited to participate. The nature of the admission diagnosis was not an exclusion criterion, only whether they were deemed to be medically stable (based on the Early Warning Score comprising heart rate and blood pressure readings). The sample included patients who were community-dwellers or living in institutional care. Patients aged <18 years who refused consent for the assessment, who were off the ward at the time of assessment (e.g., on day leave or attending another facility), who were actively dying (requiring privacy), or who were in a critical, paediatric, or maternity care setting, e.g., those in the Cardiac Care Unit, Intensive Care Unit (ICU), High-Dependency Unit or labour ward, were excluded. Where unsuitable due to advanced cognitive impairment or physical disability, verbal assent was sought. Ethics approval was obtained in advance from the local ethics committee of Galway University Hospitals (reference number C.A. 1429).

### 2.2. Sampling and Data Collection

Screening and subsequent assessment were conducted using a two-step approach. It began in the afternoon (from 16.00 on the study day) and where possible all data were collected within 24 h. Within the first hour, written information was provided to each patient explaining the nature of the study and its purpose as a hospital-wide quality improvement exercise as part of the introduction of a frailty pathway in the hospital. Informed written consent was obtained in order to assess and follow-up patient outcomes, including by chart or electronic healthcare record review. Data collectors (study raters, *n* = 10, one per ward, who were non-consultant/Resident or Fellow-level hospital doctors) received training prior to the project and inter-rater reliability (IRR) was tested on a proportion of patients on each ward, *n* = 52 in total, yielding an IRR of *r* = 0.705.

All patients were initially pre-screened using the Clinical Frailty Scale (CFS) [33]. This non-invasive, subjective, and global pictorial scale was used for a brief screening instrument and was scored from the end of bed after discussion with nursing staff on the ward. It is scored from one to nine; ranging from a score of one for ‘very fit’ to nine for ‘terminally ill’ [33]. Those patients scoring 4 or more on the CFS, indicating possible very mild frailty [34] or greater (minimum pre-frail), were then assessed with the CGA. The CFS is a widely validated scale that has been used clinically to stratify patients based on a global assessment using routine clinical assessment in hospital [23,35]. Once consent had been obtained, these trained study raters assessed each patient’s frailty status using CGA. This included two validated frailty questionnaires: the Programme of Research to Integrate Services for the Maintenance of Autonomy 7 (PRISMA-7) [36] and the FRAIL Scale [37]. Patients were also asked for information on their mobility (walking and transfer), and on their basic (dressing and washing) and instrumental (ability to perform more complex tasks, including housework) activities of daily living (ADL). Once the survey was completed, additional information to support the diagnosis was obtained from patients’ drug cards and medical charts, including details on the admitting diagnosis, baseline demographic data, diagnostic and medication lists, and available cognitive and functional information, including their requirement for home help and ability to perform ADL. This information was used to compare and support the results of the CGA. Consent was obtained from patients for this comparison. Only a study doctor examined these data. A collateral was obtained from nursing staff or family where required, again with patient consent. If it was required to provide additional clarification or where a patient was discharged within 24 h, an additional phone call was made to the patient. All data were then collated and reviewed by a consultant Geriatrician, an expert in frailty, to determine their frailty status and identify any contradictions in the data. This physician designed the study and provided training to the raters but was independent of the data collection. Based on this process, a final diagnosis was reached on current and baseline frailty (non-frail, pre-frail, or frail). If diagnosed as frail, patients were further classified as physically [38], cognitively [39] or socially [40] frail, based on accepted definitions.

### 2.3. Measures and Outcomes

In addition to the CFS, the PRISMA-7 questionnaire and FRAIL Scale were used to categorise patients. The PRISMA-7 questionnaire is a self-administered survey containing seven yes–no questions to identify frailty. One point is scored for each of the seven questions and a score of three or more supports a diagnosis of frailty. The PRISMA-7 questions cover age, gender, general health, activities, and social supports [36]. The FRAIL Scale incorporates five questions to predict frailty that ask about fatigue, resistance, ambulation, illnesses, and loss of weight [37]. Scores range from zero (not frail) to five (most frail); pre-frailty is indicated by a score of 1 or 2 and frailty by a score >2 [37]. Follow-up outcome data at one year were obtained through multiple sources, including the hospital-based electronic Patient Administration System and data from the Local Placement Forum. These outcomes included one-year mortality and long-term care (LTC; nursing home) admission, LOS, and 30 and 90-day re-admission rates from discharge. Prolonged LOS was taken as an admission duration of >7 days, due to the median LOS in Irish public sector hospitals being 6 days (average 5.6) in 2016 [41]. Admission to LTC was obtained from the county’s Local Placement Forum, excluding those already resident in a nursing home or who had died prior to discharge.

### 2.4. Statistical Analysis

Simple descriptive and correlation statistics were used to describe the prevalence of frailty in hospital and the relationships between measures of frailty, comparing baseline status to current status. Quantile–quantile plots were used to assess the normality of data, finding that the majority had a non-normal distribution. On this basis, non-parametric tests were used. Spearman’s correlation coefficient was used to assess IRR, while the Mann–Whitney U test was used to compare independent samples. The Chi-squared test compared distributions, and binary logistic and linear regressions were used to explore the strength of the relationships between variables. Binary logistic regression was used when the dependent variable was dichotomised such as for mortality (dead = yes or no). Multicollinearity among independent variables was examined using variance inflation factor analysis, removing variables from models where values exceeded >10. Linear regression was used when the outcome was continuous such as for LOS in days. Odds ratios (OR) with 95% confidence intervals (CI) were presented following adjustment for predictor variables. All data were analysed with SPSS V24.0 (Chicago, IL, USA) and R version 4.2.2 (2022-10-31)—“Innocent and Trusting” (R Core Team, 2022).

## 3. Results

**Figure 1:**
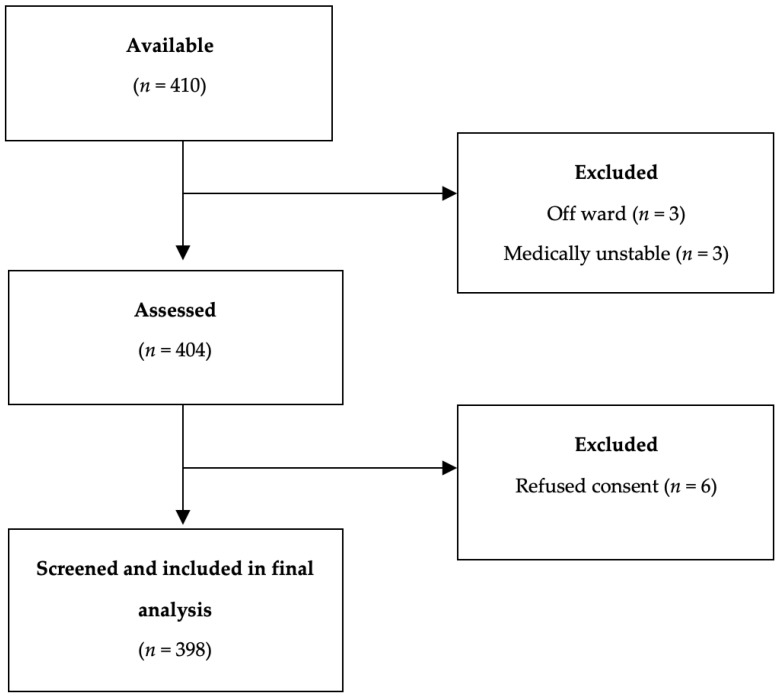
Participant flow diagram.

Of the 410 inpatients available, 398 were screened for frailty and included in this analysis. Those excluded (*n* = 12) had a median age of 71 years and 50% were male; six inpatients were excluded as they were deemed too medically unwell or were absent from the ward, and six inpatients declined to participate. A flow diagram is presented in Figure 1. The median age of the remaining sample was 70 years, interquartile (IQR) ±26 (mean 65 years) and 56% were male. In all, only 4% (16/398) of participants were currently resident in a nursing home; the remainder were community-dwellers. The median LOS at review was 8 (±17) days, while the total LOS was 8 (±17) days. Polypharmacy was common (76%). The median number of medications prescribed was seven, with 29% of participants taking at least five medications. Review of the notes suggested that 13% of participants had cognitive impairment and 8% had delirium.

Following CGA, the overall point prevalence of frailty was 30%; 14% of participants were considered pre-frail. There was a clear gradient effect with frailty prevalence lowest in those aged 18–49 (2%) and highest (44%) in those aged ≥65 years. Age was moderately correlated with frailty status based on CGA (*r* = 0.49, *p* < 0.001), with 49% of those aged ≥70 reported as frail following CGA. Patients identified as frail were older than patients without frailty (median 80 versus 64 years, *p* < 0.001) and those classified as pre-frail (median 80 versus 75 years, *p* = 0.002). Those with frailty had higher rates of polypharmacy, cognitive impairment, and delirium. These details are presented in Table 1. Based on the CGA, 22%, 12%, and 7% of participants were deemed to be physically, cognitively, or socially frail, respectively.

**Figure 2:**
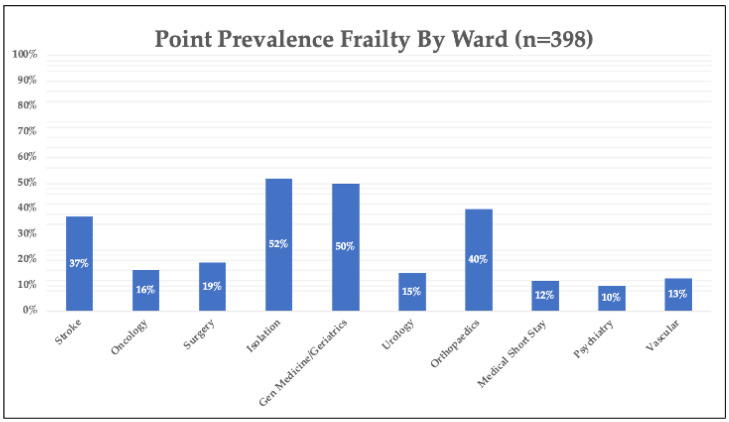
Cross-sectional prevalence of frailty (%) according to ward type based on a comprehensive geriatric assessment.

Statistically significantly more patients were classified as at least mildly frail (score ≥5/9) on the CFS at review in hospital compared to their pre-admission baseline: 45% (179/398) versus 30% (121/398), respectively; the median CFS score pre-admission was 3/9 (±3) versus 4/9 (±4) (*p* < 0.001). A smaller number of participants were pre-frail (very mildly frail) on the CFS (score of 4/9): 12% (48/398). Of those aged ≥65 years, 45% were frail based on their pre-morbid CFS and 60% were frail based on their current CFS score, using a cut-off score of ≥5/9. Age had a stronger correlation with baseline CFS scores (*r* = 0.60) than with current scores (*r* = 0.54). Median PRISMA-7 and FRAIL Scale scores were 4 (±3) and 2 (±2), respectively; the prevalences of frailty as measured by these tools were 29% and 15%, respectively. For those aged ≥65 years, 42% were identified as frail using the PRISMA-7 and 21% were identified as frail using the FRAIL Scale. Frailty was found throughout the study but was most common on general internal medicine and orthopaedic wards and lowest on psychiatric and all other surgical wards (general, vascular, and urology), see Figure 2.

At the one-year follow-up, 27% (*n* = 108) of inpatients were dead. Those aged ≥65 years had a higher mortality rate of 34% (identical to the rate for those ≥70). Patients classified as frail based on the CGA were statistically significantly more likely to be dead at the one-year follow-up (*p* < 0.001). Inpatient mortality was lower at 6% overall, and while it was higher in those identified as frail, this was not statistically significant. At one-year follow-up, 6% of non-institutionalised adults had been admitted to a nursing home. Similarly, older patients (≥65) had a higher rate (9%) of nursing home admission and those who were frail were statistically more likely to be institutionalised (*p* < 0.001). Almost one-quarter (24%) of patients who were frail were re-admitted within 90 days of discharge after the index admission, a prevalence that was significantly greater than that for those who were non-frail (18%, *p* = 0.04). The comparisons of outcomes between those identified as frail versus non-frail are presented in Table 2. After adjustment for age and biological sex, frailty remained an independent predictor of prolonged LOS (≥7 days), with OR 1.71 (95% CI: 1.01–2.88, *p* = 0.0453); one-year mortality, with OR 2.08 (95% CI: 1.23–3.50, *p* = 0.006); and one-year institutionalisation, with OR 9.03 (95% CI: 2.45–33.2, *p* = 0.001). After adjusting for age and sex, being frail was associated with an increase of 30.5 days in LOS (95% CI: 8.84–52.1 days, *p* = 0.006) over those who were non-frail. There was no statistically significant association for re-admission (at 30 or 90 days) after adjustment.

## 4. Discussion

This study examining the cross-sectional prevalence of frailty in a large-regional-acute university hospital in Ireland found that the point prevalence of frailty, based on a CGA, among all inpatients aged ≥18 years was 30%. The current rate varied according to the frailty measure used, ranging from as low as 15% with a measure of physical frailty, the FRAIL Scale, and to 45% with a more global measure of frailty, the CFS. Examination of older adults (i.e., those aged ≥65) found that the overall current point prevalence increased to 44% based on the CGA, to 60% using the CFS, and to 21% applying the FRAIL Scale.

These results compare favourably with international studies conducted across a range of hospitals across the world. In a systematic review and meta-analysis, based on data from 21 countries across five continents, and using a variety of frailty measures exclusively among older adults (minimum age aged ≥ 65 years), the overall pooled estimate of frailty prevalence was 47.4% with an estimate of 25.8% for pre-frailty [11]. In Austria, the prevalence of frailty among randomly sampled patients who were ≥65 and scheduled for discharge within two weeks (*n* = 133) was 54.1% based on the SHARE-FI tool [42]. In Vietnam, the prevalence of frailty in a cross-sectional study of 461 consecutive admissions aged ≥60 was 31.9% using the Reported Edmonton Frail Scale and 35.4% using the Fried Physical Frailty Phenotype [43]. These studies reflect the marked heterogeneity reported in and between meta-analyses [3,11], which is not related to national economic measures but instead to patient characteristics and frailty measures [11].

Fewer studies have examined cross-sectional data and presented point prevalence based on knowledge of an individual’s pre-admission frailty status. Our own study group has found a similar rate in a single hospital using the CFS (score ≥ 5) as part of a separate study examining continence in a tertiary referral hospital, conducted in 2017, which reported that 25% of inpatients were frail prior to admission [44]. The point prevalence was markedly higher for current frailty status, where 52% scored ≥5 on the CFS, again similar to this study, which found that 45% of participants were at least mildly frail. The diagnosis of frailty was not, however, based on CGA, unlike our current study. Also in Ireland, a smaller, cross-sectional study of 233 inpatients in the South of the country, which similarly defined frailty using the CFS (score ≥ 5) rather than a detailed CGA, found that 39.5% of participants were frail, with a higher prevalence (52%) in those aged ≥65 [45]. A similar, albeit higher, prevalence was found in a single hospital in New Zealand, where 49% of inpatients were classified as frail on the (reported) Edmonton Frail Scale when referencing the time of admission rather than pre-admission status [46]. In our study, the lower prevalence of frailty (30%) likely reflects the more detailed and accurate approach to identifying true frailty using CGA [47] and the more nuanced reporting of prior frailty status rather than an individual’s current status, which is reflected by instruments such as the Reported Edmonton Frail Scale [48]. Indeed, heterogeneity related to differences in frailty screening instruments and definitions to assess frailty contribute to the variations in prevalence reported across settings [12].

To date, relatively few studies have reported frailty prevalence in hospital according to ward or unit type. In our study, we found that the prevalence of frailty was highest among those admitted to general medical/geriatric medicine (50%) and orthopaedic wards (40%) and lowest in psychiatric and surgical wards (general, vascular, and urology). While fewer wards were available, this trend was similar to the study in the South of Ireland [45]. In a recent systematic review, the pooled prevalence of frailty in general medicine wards was 59.3% compared to 66.5% on geriatric medicine wards and just 30.6% on surgical wards with statistically significant differences between wards [12], though the authors note that there was marked heterogeneity between studies.

Unlike these other studies, we followed patients for a year after the cross-sectional assessment to examine a range of healthcare outcomes. Here we showed that, among an inpatient population, frailty was associated with increased odds of prolonged LOS, one-year mortality, and one-year risk of institutionalisation in a nursing home. These associations remained significant after adjustment for age and sex. Frailty did not significantly influence the risk of re-admission to hospital. These results are similar to findings across other studies, including a large meta-analysis [3]. For example, in the United Kingdom (UK) a large retrospective study of patients aged ≥75 years who were admitted consecutively to a large university hospital found that the prevalence of frailty, based on a CFS score ≥ 5 administered within three days, was an independent predictor of LOS and mortality [25]. Other factors associated with adverse outcomes in this study included delirium, which was independently associated with inpatient mortality, and cognitive impairment, which was itself associated with nursing home admission at one year. Delirium has been established as a risk factor for increased inpatient mortality [49], particularly among critically unwell older adults [50]. Cognitive decline among inpatients is well established as increasing the risk of admission to long-term nursing care from acute hospital care [51].

We were also able to compare pre-admission and cross-sectional frailty scores. We showed that frailty status as measured by global frailty measures (i.e., the CFS) appears to worsen significantly during admission, which is likely reflecting acute illness and may not reflect a patient’s true frailty level. This usually relates to a change in functional status, which contributes to the diagnosis of frailty across a range of measures. An Australian study examining surgical and orthopaedic patients aged ≥70 found significant increases in mean Frailty Index scores when comparing pre-admission and current statuses [52]. Both were associated with one-year mortality [52].

While most researchers suggest that frailty is exclusively a condition associated with ageing and, hence, older people, there are a growing number of studies examining frailty in younger adults, including in hospital [53,54,55]. A study by Gordon et al. in 2023 suggests that frailty is common among hospital inpatients in Australia aged 18–49, with over one-quarter 26.6% of inpatients reported as frail using a Frailty Index [55]. While it was not associated with mortality, it increased LOS [55]. In our study examining frailty among general inpatients aged ≥18, we also showed that frailty is found in patients aged <65 years but at a much lower rate than in these studies: 2% versus 26.6% in Australian hospitals [55]. This may again be explained by the heterogeneity between the studies comparing frailty in acute care settings [56].

In this paper, we explored frailty in a large sample of Irish inpatients and have shown the impact of this diagnostic label on outcomes up to one year after admission. While there is a growing rationale for screening for frailty and understanding its prevalence in acute care settings, there remain unanswered questions. In particular, it is not clear (1) whether there are negative consequences to the diagnosis of frailty in hospital (e.g., therapeutic nihilism and the denial of care) (2), which screening instruments have the greatest diagnostic accuracy, or (3) what the trajectories of frailty in hospital look like and how an admission affects these [56]. Further, given the clear demonstration of statistically significant differences between recent pre-admission frailty status and current point prevalence of frailty in hospital, there is a need to separate baseline vulnerability from the effects of the acute illness [57].

### Strengths, Limitations, and Areas for Further Research

Strengths of this study include the fact that all inpatients aged ≥18 were eligible for inclusion. The diagnosis of frailty, based on a CGA, was robust [47] in comparison to many similar studies, and pre-admission as well as current cross-sectional frailty status were considered. This study employed a two-step screening and assessment process to identify frailty. This approach has been used before in research studies stratifying frailty [58] and is recommended as the optimal approach to categorising patients in epidemiological studies [59]. A limitation is that data on past medical history, including a current diagnosis of cognitive impairment, delirium, or polypharmacy, were based on a review of the medical notes, which may have meant that the diagnoses were under-reported in this study (due to incomplete data), potentially introducing bias and reducing generalisability. Every effort was made to obtain a collateral from nursing staff or family, where this was deemed necessary or available. While individually valid and widely published frailty instruments, including the CFS, FRAIL Scale, and PRISMA-7, were used as part of the CGA, no standardised approach to CGA is available, potentially further reducing generalisability. Further, while their predictive accuracy for adverse outcomes such as mortality is well established, the diagnostic accuracy of CGA for frailty in this setting is less clear. Additional research is needed to examine the diagnostic accuracy of frailty instruments in acute care measured against CGA as a ‘gold standard’. However, scales were selected for practical considerations and were only used to support the diagnosis made by the Geriatrician in the context of available information. They did not act as substitutes for the CGA. The decision to exclude patients in ICU and other critical care settings likely led to an underestimation of the frailty prevalence in hospital, with rates shown to be approximately 30% in these settings [60]. These patients were excluded for practical reasons given, the challenges of performing frailty assessments in these patients and the different assessments required. For example, the CFS has uncertain reliability among patients in critical care [61]. Finally, these data were collected in 2016 and an initial and incomplete analysis was presented in abstract form at the Irish Gerontological Society meeting in 2017. Follow-up outcome data were obtained in 2019. Due to the intervening relocation of key staff and disruption caused by the subsequent COVID-19 pandemic in 2020–2023, this work was not submitted for formal publication until 2023, potentially reducing the impact or veracity of these data. However, this is unlikely given the epidemiological nature of the study and the robust approach to determining frailty by CGA, which remains the most widely accepted standard. Nevertheless, given the increasing frailty of ageing populations, the authors caution that the true point prevalence may be even higher. Further studies in different sites both nationally and internationally using a similar approach to that utilised in this study are now needed to confirm and update these results. Such an approach has been utilised for other conditions among inpatients, including the cross-sectional prevalence of delirium across Italian hospitals on “delirium day” in 2015 [62]; a similar approach could be taken for frailty, i.e., a national “frailty day”, which could then be repeated over time to monitor for change. Understanding the changes in the prevalence of frailty over time has important implications for the development of frailty pathways in Ireland and the development of integrated care linking acute and community care [63,64,65]. It is also relevant in a European context [66] and future studies could compare results across countries and regions. Further study is now needed to examine the merits of more routine and indeed “real time” continuous frailty screening in acute care, and to examine whether the instigation of specific interventions based on CGA in hospital can mitigate adverse outcomes among inpatients with frailty [56], irrespective of age.

## 5. Conclusions

Understanding the epidemiology, including the point prevalence of frailty in acute care settings, is important in order to better align resources such as CGA, an important risk assessment for hospitalised patients, and for cohorting patients appropriately on wards where this can be delivered. This study highlights the fact that frailty is highly prevalent among acutely hospitalised patients as defined by CGA; one-third of all adult inpatients and 44% of older patients were frail in this study. This study reaffirms that frailty is associated with an increased prevalence of hospital LOS, one-year mortality, and one-year risk of institutionalisation amongst hospitalised inpatients. The development of frailty clinical care pathways in acute care is recommended to address the poor prognosis associated with a diagnosis of frailty in this setting. As this study is the first study in Ireland and one of the first to our knowledge to use CGA to characterise frailty prevalence across a hospital, further study both nationally and internationally is recommended to confirm these findings.

## Figures and Tables

**Table 1:** Characteristics of hospital inpatients (*n* = 398) screened and assessed for frailty and comparison of those classified as frail and non-frail (robust and pre-frail).

Predictor	Total (*n* = 398) Median (Q3–Q1 = +/−IQR)	Frail (*n* = 120; 30%) Median (Q3–Q1 = +/−IQR)	Non-Frail (*n* = 278; 70%) Median (Q3–Q1 = +/−IQR)	*p* Value
Age (Years)	69.5 (80–54 = ±26)	80 (86–75 = ±9)	64 (74–44 = ±30)	*p* < 0.001 *
Sex (% Male)	56%	49%	59%	*p* = 0.08
Medications (Number, any)	7 (10–5 = ±5)	8 (11–2 = ±9)	6 (9–4 = ±5)	*p* = 0.002 *
Polypharmacy (% with ≥5)	29%	55%	18%	*p* = 0.014 *
Cognitive impairment (% documented)	13%	36%	3%	*p* < 0.001 *
Delirium (% documented)	8%	19%	3%	*p* = 0.005 *
CFS (Baseline)	3 (5–2 = ±3)	6 (6–5 = ±1)	2 (3–2 = ±1)	*p* < 0.001 *
CFS (Current)	4 (6–2 = ±4)	6 (7–6 = ±1)	3 (4–2 = ±2)	*p* < 0.001 *
FRAIL Scale	2 (3–1 = ±2)	3 (4–2 = ±2)	1 (2–1 = ±1)	*p* < 0.001 *
PRISMA-7	4 (5–2 = ±3)	5 (6–5 = ±1)	2 (3–2 = ±1)	*p* < 0.001 *

CFS—Clinical Frailty Scale; PRISMA-7—Programme of Research to Integrate Services for the Maintenance of Autonomy 7; IQR—Interquartile range; *—Statistically significant.

**Table 2:** Outcomes of hospital inpatients (n = 398) screened and assessed for frailty compared between those classified as frail and non-frail (robust and pre-frail).

Predictor	Total Median (Q3–Q1 = +/−IQR)	Frail Median (Q3–Q1 = +/−IQR)	Non-Frail Median (Q3–Q1 = +/−IQR)	*p* Value
LOS (days)	8 (21–4 = ±17)	12 (31–5 = ±26)	6 (15-3 = ±12)	*p* < 0.001 *
Prolonged LOS (% median ≥7 days)	54%	66%	48%	*p* = 0.001 *
30-day re-admission rate (%)	12%	18%	9%	0.03 *
90-day re-admission rate (%)	16%	24%	13%	0.04 *
Inpatient mortality (% prior to discharge)	6%	10%	4%	0.07
Mortality (One-year %)	27%	43%	20%	*p* < 0.001 *
Nursing home admission (One-year %, excluding existing residents)	6%	16%	1%	*p* < 0.001 *

LOS—Length of Stay; IQR—Interquartile range; *—Statistically significant.

## Data Availability

Data are available on request.

## References

[1] Clegg A., Young J., Iliffe S., Rikkert M.O., Rockwood K. (2013). Frailty in elderly people. Lancet.

[2] Khandelwal D., Goel A., Kumar U., Gulati V., Narang R., Dey A.B. (2012). Frailty is associated with longer hospital stay and increased mortality in hospitalized older patients. J. Nutr. Health Aging.

[3] Boucher E.L., Gan J.M., Rothwell P.M., Shepperd S., Pendlebury S.T. (2023). Prevalence and outcomes of frailty in unplanned hospital admissions: A systematic review and meta-analysis of hospital-wide and general (internal) medicine cohorts. EClinicalMedicine.

[4] Vermeiren S., Vella-Azzopardi R., Beckwee D., Habbig A.K., Scafoglieri A., Jansen B., Bautmans I., Verté D., Beyer I., Petrovic M. (2016). Frailty and the prediction of negative health outcomes: A meta-analysis. JAMDA.

[5] Hartley P., Adamson J., Cunningham C., Embleton G., Romero-Ortuno R. (2017). Clinical frailty and functional trajectories in hospitalized older adults: A retrospective observational study. Geriatr. Geront. Int..

[6] Heppenstall C., Wilkinson T.J., Hanger H.C., Keeling S. (2011). Factors related to care home admission in the year following hospitalisation in frail older adults. Age Ageing.

[7] Sezgin D., O’Donovan M., Woo J., Bandeen-Roche K., Liotta G., Fairhall N., Rodríguez-Laso A., Apóstolo J., Clarnette R., Holland C. (2022). Early identification of frailty: Developing an international delphi consensus on pre-frailty. Arch. Gerontol. Geriatr..

[8] Galluzzo L., O’Caoimh R. (2018). Epidemiology, surveillance and population screening of frailty. Results from the systematic reviews of the European Joint Action ADVANTAGE. Preface. Ann. Dell’Istituto Super. Sanità.

[9] Liotta G., Ussai S., Illario M., O’Caoimh R., Cano A., Holland C., Roller-Winsberger R., Capanna A., Grecuccio C., Ferraro M. (2018). Frailty as the Future Core Business of Public Health: Report of the Activities of the A3 Action Group of the European Innovation Partnership on Active and Healthy Ageing (EIP on AHA). Int. J. Environ. Res. Public. Health.

[10] Gilardi F., Scarcella P., Proietti M.G., Capobianco G., Rocco G., Capanna A., Mancinelli S., Marazzi M.C., Palombi L., Liotta G. (2018). Frailty as a predictor of mortality and hospital services use in older adults: A cluster analysis in a cohort study. Eur. J. Pub. Health.

[11] Liotta G., Gilardi F., Orlando S., Rocco G., Proietti M.G., Asta F., De Sario M., Michelozzi P., Mancinelli S., Palombi L. (2019). Cost of hospital care for the older adults according to their level of frailty. A cohort study in the Lazio region, Italy. PloS ONE.

[12] Doody P., Asamane E.A., Aunger J.A., Swales B., Lord J.M., Greig C.A., Whittaker A.C. (2022). The prevalence of frailty and pre-frailty among geriatric hospital inpatients and its association with economic prosperity and healthcare expenditure: A systematic review and meta-analysis of 467,779 geriatric hospital inpatients. Ageing Res. Rev..

[13] Cechinel C., Lenardt M.H., Rodrigues J.A.M., Binotto M.A., Aristides M.M., Kraus R. (2022). Frailty and delirium in hospitalized older adults: A systematic review with meta-analysis. Rev. Latino-Americana Enferm..

[14] Hubbard R.E., Peel N.M., Samanta M., Gray L.C., Mitnitski A., Rockwood K. (2017). Frailty status at admission to hospital predicts multiple adverse outcomes. Age. Ageing.

[15] Cunha A.I.L., Veronese N., de Melo Borges S., Ricci N.A. (2019). Frailty as a predictor of adverse outcomes in hospitalized older adults: A systematic review and meta-analysis. Ageing Res. Rev..

[16] Keeble E., Roberts H.C., Williams C.D., Van Oppen J., Conroy S.P. (2019). Outcomes of hospital admissions among frail older people: A 2-year cohort study. Br. J. Gen. Pract..

[17] Gilbert T., Neuburger J., Kraindler J., Keeble E., Smith P., Ariti C., Arora S., Street A., Parker S., Roberts H.C. (2018). Development and validation of a Hospital Frailty Risk Score focusing on older people in acute care settings using electronic hospital records: An observational study. Lancet.

[18] Rezaei-Shahsavarloo Z., Atashzadeh-Shoorideh F., Gobbens R.J.J., Ebadi A., Ghaedamini Harouni G. (2020). The impact of interventions on management of frailty in hospitalized frail older adults: A systematic review and meta-analysis. BMC Geriatr..

[19] Ellis G., Gardner M., Tsiachristas A., Langhorne P., Burke O., Harwood R.H., Conroy S.P., Kircher T., Somme D., Saltvedt I. (2017). Comprehensive geriatric assessment for older adults admitted to hospital. Cochrane. Database. Syst. Rev..

[20] Partridge J.S., Harari D., Dhesi J.K. (2012). Frailty in the older surgical patient: A review. Age. Ageing.

[21] Soong J., Poots A.J., Scott S., Donald K., Woodcock T., Lovett D., Bell D. (2015). Quantifying the prevalence of frailty in English hospitals. BMJ Open.

[22] Kennelly S.P., Drumm B., Coughlan T., Collins R., O’Neill D., Romero-Ortuno R. (2014). Characteristics and outcomes of older persons attending the emergency department: A retrospective cohort study. QJM.

[23] Gregorevic K.J., Hubbard R.E., Katz B., Lim W.K. (2016). The clinical frailty scale predicts functional decline and mortality when used by junior medical staff: A prospective cohort study. BMC Geriatr..

[24] Joosten E., Demuynck M., Detroyer E., Milisen K. (2014). Prevalence of frailty and its ability to predict in hospital delirium, falls, and 6-month mortality in hospitalized older patients. BMC Geriatr..

[25] Wallis S.J., Wall J., Biram R.W., Romero-Ortuno R. (2015). Association of the clinical frailty scale with hospital outcomes. QJM.

[26] Theou O., Squires E., Mallery K., Lee J.S., Fay S., Goldstein J., Armstrong J.J., Rockwood K. (2018). What do we know about frailty in the acute care setting? A scoping review. BMC Geriatr..

[27] O’Caoimh R., Cooney M.T., Cooke J., O’Shea D. (2019). The challenges of using the Hospital Frailty Risk Score. Lancet.

[28] Walston J., Buta B., Xue Q.L. (2018). Frailty Screening and Interventions: Considerations for Clinical Practice. Clin. Geriatr. Med..

[29] Liu X., Le M.K., Lim A.Y.C., Koh E.J., Nguyen T.N., Malik N.A., Lien C.T.C., Lee J.E., Au L.S.Y., Low J.A.Y.H. (2022). Perspectives on frailty screening, management and its implementation among acute care providers in Singapore: A qualitative study. BMC Geriatr..

[30] Elliott A., Phelps K., Regen E., Conroy S.P. (2017). Identifying frailty in the Emergency Department-feasibility study. Age Ageing.

[31] Moloney E., O’Donovan M.R., Sezgin D., McGrath K., Timmons S., O’Caoimh R. (2023). Frailty Knowledge, Use of Screening Tools, and Educational Challenges in Emergency Departments in Ireland: A Multisite Survey. J. Emerg. Nurs..

[32] Harnett P.J., Kennelly S., Williams P. (2020). A 10 Step Framework to Implement Integrated Care for Older Persons. Ageing Int..

[33] Rockwood K., Song X., MacKnight C., Bergman H., Hogan D.B., McDowell I., Mitnitski A. (2005). A global clinical measure of fitness and frailty in elderly people. CMAJ.

[34] Rockwood K., Theou O. (2020). Using the Clinical Frailty Scale in Allocating Scarce Health Care Resources. Can. Geriatr. J..

[35] Church S., Rogers E., Rockwood K., Theou O. (2020). A scoping review of the Clinical Frailty Scale. BMC Geriatr..

[36] Raîche M., Hébert R., Dubois M.F. (2008). PRISMA-7: A case-finding tool to identify older adults with moderate to severe disabilities. Arch. Gerontol. Geriatr..

[37] Morley J.E., Malmstrom T.K., Miller D.K. (2012). A simple frailty questionnaire (FRAIL) predicts outcomes in middle aged African Americans. J. Nutr. Health Aging..

[38] Fried L.P., Tangen C.M., Walston J., Newman A.B., Hirsch C., Gottdiener J., Seeman T., Tracy R., Kop W.J., Burke G. (2001). Frailty in older adults: Evidence for a phenotype. J. Gerontol. A Biol. Sci. Med. Sci..

[39] Kelaiditi E., Cesari M., Canevelli M., van Kan G.A., Ousset P.J., Gillette-Guyonnet S., Ritz P., Duveau F., Soto M.E., Provencher V. (2013). Cognitive frailty: Rational and definition from an (I.A.N.A./I.A.G.G.) international consensus group. J. Nutr. Health Aging.

[40] Makizako H., Shimada H., Tsutsumimoto K., Lee S., Doi T., Nakakubo S., Hotta R., Suzuki T. (2014). Social Frailty in Community-Dwelling Older Adults as a Risk Factor for Disability. JAMDA.

[41] (2018). Eurostat. Hospital Discharges and Length of Stay Statistics.

[42] Dorner T.E., Luger E., Tschinderle J., Stein K.V., Haider S., Kapan A., Lackinger C., Schindler K.E. (2014). Association between nutritional status (MNA®-SF) and frailty (SHARE-FI) in acute hospitalised elderly patients. J Nutr. Health Aging.

[43] Vu H.T.T., Nguyen T.X., Nguyen T.N., Nguyen A.T., Cumming R., Hilmer S., Pham T. (2017). Prevalence of frailty and its associated factors in older hospitalised patients in Vietnam. BMC Geriatr..

[44] Condon M., Mannion E., Molloy D.W., O’Caoimh R. (2019). Urinary and Faecal Incontinence: Point Prevalence and Predictors in a University Hospital. Int. J. Environ. Res. Public. Health.

[45] O’Donnell D., O’Mahony A., Doyle M., O’Gorman M., O’Donoghue A., O’Halloran A., Mulcahy R., Pope G., Cooke J. (2022). Point Prevalence of Frailty and Cognitive Impairment Exceeds the Capacity of a Single Ward—Specialist Geriatric Wards to lead Best Practice. Irish. Med. J..

[46] Richards S.J.G., D’Souza J., Pascoe R., Falloon M., Frizelle F.A. (2019). Prevalence of frailty in a tertiary hospital: A point prevalence observational study. PloS ONE.

[47] Lee H., Lee E., Jang I.Y. (2020). Frailty and Comprehensive Geriatric Assessment. J. Korean Med. Sci..

[48] Hilmer S.N., Perera V., Mitchell S., Murnion B.P., Dent J., Bajorek B., Matthews S., Rolfson D.B. (2009). The assessment of frailty in older people in acute care. Australas. J. Ageing.

[49] Al Huraizi A.R., Al-Maqbali J.S., Al Farsi R.S., Al Zeedy K., Al-Saadi T., Al-Hamadani N., Al Alawi A.M. (2023). Delirium and Its Association with Short- and Long-Term Health Outcomes in Medically Admitted Patients: A Prospective Study. J. Clin. Med..

[50] Sahle B.W., Pilcher D., Litton E., Ofori-Asenso R., Peter K., McFadyen J., Bucknall T. (2022). Association between frailty, delirium, and mortality in older critically ill patients: A binational registry study. Ann. Intensiv. Care.

[51] Bu F., Rutherford A. (2019). Dementia, home care and institutionalisation from hospitals in older people. Eur. J. Ageing.

[52] Lin H.S., Watts J.N., Peel N.M., Hubbard R.E. (2016). Frailty and post-operative outcomes in older surgical patients: A systematic review. BMC Geriatr..

[53] Hewitt J., Carter B., McCarthy K., Pearce L., Law J., Wilson F.V., Tay H.S., McCormack C., Stechman M.J., Moug S.J. (2019). Frailty predicts mortality in all emergency surgical admissions regardless of age. An observational study. Age Ageing.

[54] Spiers G.F., Kunonga T.P., Hall A., Beyer F., Boulton E., Parker S., Bower P., Craig D., Todd C., Hanratty B. (2021). Measuring frailty in younger populations: A rapid review of evidence. BMJ Open.

[55] Gordon E.H., Peel N.M., Hubbard R.E., Reid N. (2023). Frailty in younger adults in hospital. QJM.

[56] Hogan D.B., Maxwell C.J., Afilalo J., Arora R.C., Bagshaw S.M., Basran J., Bergman H., Bronskill S.E., Carter C.A., Dixon E. (2017). A Scoping Review of Frailty and Acute Care in Middle-Aged and Older Individuals with Recommendations for Future Research. Can. Geriatr. J..

[57] Hilmer S., Hubbard R.E. (2022). Where next with frailty risk scores in hospital populations?. Age Ageing.

[58] Jansen-Kosterink S., Van Velsen L., Frazer S., Dekker-van Weering M., O’Caoimh R., Vollenbroek-Hutten M. (2019). Identification of community-dwelling older adults at risk of frailty using the PERSSILAA screening pathway: A methodological guide and results of a large-scale deployment in the Netherlands. BMC Public. Health.

[59] Rodríguez-Laso Á., O’Caoimh R., Galluzzo L., Carcaillon-Bentata L., Beltzer N., Macijauskiene J., Albaina Bacaicoa O., Ciutan M., Hendry A., López-Samaniego L. (2018). Population screening, monitoring and surveillance for frailty: Three systematic reviews and a grey literature review. Ann. Ist. Super. Sanita..

[60] Muscedere J., Waters B., Varambally A., Bagshaw S.M., Boyd J.G., Maslove D., Sibley S., Rockwood K. (2017). The impact of frailty on intensive care unit outcomes: A systematic review and meta-analysis. Intens. Care. Med..

[61] Pugh R.J., Ellison A., Pye K., Subbe C.P., Thorpe C.M., Lone N.I., Clegg A. (2018). Feasibility and reliability of frailty assessment in the critically ill: A systematic review. Crit. Care..

[62] Bellini G., Morandi A., Di Santo S.G., Mazzone A., Cherubini A., Mossello E., Bo M., Bianchetti A., Rozzini R., Zanetti E. (2016). "Delirium Day": A nationwide point prevalence study of delirium in older hospitalized patients using an easy standardized diagnostic tool. BMC Med..

[63] Conroy S.P., Stevens T., Parker S.G., Gladman J.R. (2011). A systematic review of comprehensive geriatric assessment to improve outcomes for frail older people being rapidly discharged from acute hospital: ’interface geriatrics’. Age Ageing.

[64] Ní Shé É., McCarthy M., O’Donnell D., Collins O., Hughes G., Salter N., Cogan L., O’Donoghue C., McGrath E., O’Donovan J. (2018). The systematic approach to improving care for Frail Older Patients (SAFE) study: A protocol for co-designing a frail older person’s pathway. HRB Open Res..

[65] Barry S., Fhallúin M.N., Thomas S., Harnett P.J., Burke S. (2021). Implementing Integrated Care in Practice—Learning from MDTs Driving the Integrated Care Programme for Older Persons in Ireland. Int. J. Integr. Care..

[66] Hendry A., Vanhecke E., Carriazo A.M., López-Samaniego L., Espinosa J.M., Sezgin D., O’Donovan M., Hammar T., Ferry P., Vella A. (2019). Integrated Care Models for Managing and Preventing Frailty: A Systematic Review for the European Joint Action on Frailty Prevention (ADVANTAGE JA). Transl. Med. UniSa..

